# Off-Stream Watering Systems and Partial Barriers as a Strategy to Maximize Cattle Production and Minimize Time Spent in the Riparian Area

**DOI:** 10.3390/ani4040670

**Published:** 2014-10-29

**Authors:** Ashley A. Rawluk, Gary Crow, Getahun Legesse, Douglas M. Veira, Paul R. Bullock, Luciano A. González, Melanie Dubois, Kim H. Ominski

**Affiliations:** 1 Ducks Unlimited Canada, Calgary, T2Z 3V6, Canada; E-Mail: a_rawluk@ducks.ca; 2 Department of Animal Science, University of Manitoba, Winnipeg, R3T 2N2, Canada; E-Mails: gary.crow@umanitoba.ca (G.C.); getahun_legesse@umanitoba.ca (G.L.); 3 Pacific Agri-Food Research Centre, Agriculture and Agri-Food Canada, Agassiz, V0M 1A0, Canada; E-Mail: doug.veira@agr.gc.ca; 4 Department of Soil Science, University of Manitoba, Winnipeg, R3T 2N2, Canada; E-Mail: paul.bullock@umanitoba.ca; 5 Centre for Carbon, Water and Food, Faculty of Agriculture and Environment, The University of Sydney, Camden, NSW 2570, Australia; E-Mail: luciano.gonzalez@sydney.edu.au; 6 Agriculture and Agri-Food Canada, 2701 Grand Valley Rd, Brandon, R7A 5Y3, Canada; E-Mail: melanie.dubois@agr.gc.ca

**Keywords:** riparian area, off-stream waterer, exclusion fencing, animal performance, Canada, cattle distribution, GPS

## Abstract

**Simple Summary:**

The implementation of off-stream waterers (OSW) may reduce the amount of time cattle spend in riparian areas, thus minimizing impacts such as removal of vegetation, soil compaction, and deterioration in water quality. Furthermore, when used with natural barriers as a partial exclusion method, these management strategies may offer a cost-effective alternative to completely excluding cattle via streambank fencing. This study was conducted to determine the impact of OSW and barriers on animal performance and watering behavior. The presence of OSW had no significant effect on cow and calf weights averaged over the grazing season. Although the results were not consistent over the periods and locations, the data provided some indication of the efficacy of the natural barriers on deterring cattle from the riparian area. Cattle watered at the OSW when available, but they did not use the OSW exclusively. The observed inconsistency may, in part, be attributed to the environmental conditions present during this field trial.

**Abstract:**

A study was conducted in 2009 at two locations in Manitoba (Killarney and Souris), Canada to determine the impact of off-stream waterers (OSW) with or without natural barriers on (i) amount of time cattle spent in the 10 m buffer created within the riparian area, referred to as the riparian polygon (RP), (ii) watering location (OSW or stream), and (iii) animal performance measured as weight gain. This study was divided into three 28-day periods over the grazing season. At each location, the pasture—which ranged from 21.0 ha to 39.2 ha in size—was divided into three treatments: no OSW nor barriers (1CONT), OSW with barriers along the stream bank to deter cattle from watering at the stream (2BARR), and OSW without barriers (3NOBARR). Cattle in 2BARR spent less time in the RP in Periods 1 (*p* = 0.0002), 2 (*p* = 0.1116), and 3 (*p* < 0.0001) at the Killarney site compared to cattle in 3NOBARR at the same site. Cattle in 2BARR at the Souris site spent more time in the RP in Period 1 (*p* < 0.0001) and less time in Period 2 (*p* = 0.0002) compared to cattle in 3NOBARR. Cattle did use the OSW, but not exclusively, as watering at the stream was still observed. The observed inconsistency in the effectiveness of the natural barriers on deterring cattle from the riparian area between periods and locations may be partly attributable to the environmental conditions present during this field trial as well as difference in pasture size and the ability of the established barriers to deter cattle from using the stream as a water source. Treatment had no significant effect (*p* > 0.05) on cow and calf weights averaged over the summer period. These results indicate that the presence of an OSW does not create significant differences in animal performance when used in extensive pasture scenarios such as those studied within the present study. Whereas the barriers did not consistently discourage watering at the stream, the results provide some indication of the efficacy of the OSW as well as the natural barriers on deterring cattle from the riparian area.

## 1. Introduction

Cow/calf operators may use streams within riparian areas as a water source for livestock. Cattle are attracted to riparian areas as they provide water, forage, and shade [1]. While grazing and watering in the riparian area, cattle may contribute to the removal of vegetation, soil compaction and erosion, and degradation of water quality [2,3]. In order to minimize impacts to riparian areas, livestock producers are encouraged to adopt best management practices (BMP) that are environmentally and economically sustainable.

Exclusion fencing is a BMP that has proven to be effective [4,5]; however, it is costly, removes access to large areas of pasture, and gives the impression that cattle and riparian areas cannot be managed to exist harmoniously [6]. Off-stream waterers (OSW) are an alternative method to exclusion fencing. Previous studies have shown reductions in the amount of time spent in the riparian area or stream when an OSW is available [7,8,9,10,11]. After retrospectively evaluating the effects of rangeland BMP implementation on riparian areas using satellite imagery time series, Rigge *et al.* [12] recently reported that management practices such as cross-fencing and OSW positively affected the riparian vegetation cover. Other studies have found that cattle will drink more frequently from the OSW than the stream [10,13]. As many of these studies were carried out in small pastures, further research regarding the effectiveness of OSW is required in larger pastures, characterized by undulating topography, forested areas, and varying precipitation; all of which are features typical of pastureland located in many regions including Southern Manitoba. Use of natural barriers (e.g., fallen trees and large boulders) has been suggested as a strategy to improve the effectiveness of OSW in that they may serve to partially exclude livestock from accessing established crossing and watering locations [14]. Research examining the effectiveness of this strategy is currently limited. In order to adopt BMP such as OSW with or without the use of natural barriers, livestock producers must be assured that the implementation of these strategies will not negatively impact animal behavior (e.g., watering and grazing behavior) and performance. The objectives of this research were to explore the use of OSW in large-scale pastures in terms of animal performance and to examine the effectiveness of low-cost barriers at defined crossing and watering locations.

## 2. Experimental Section

### 2.1. Site Description

**Figure 1:**
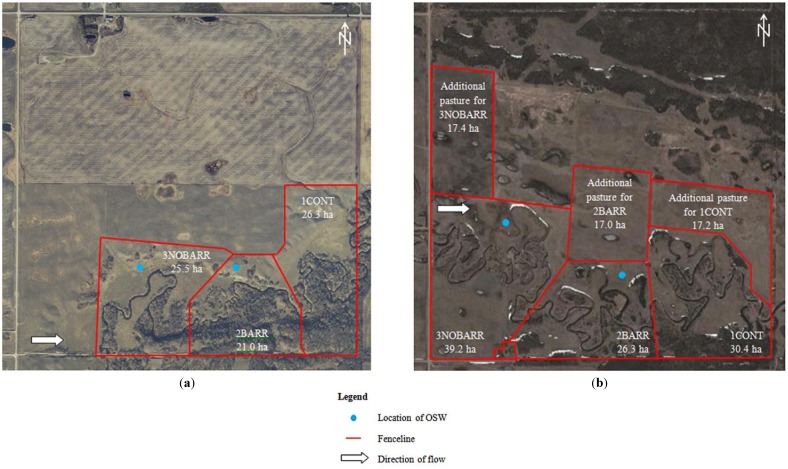
Site layout at (**a**) Killarney and (**b**) Souris locations.

The study was conducted in 2009 at two locations in south western Manitoba, Canada; one site near the town of Killarney on the Pembina River and the second near the town of Souris on Plum Creek (Figure 1). Criteria for pasture selection included: (1) continuously grazed; (2) comprised largely of native or tame species, with similar forage types; (3) similar carrying capacity and stocking density of approximately 25 cow/calf pairs; and (4) adjacent to a stream, which flowed for the duration of the trial, with pre-existing fencing around the pasture, and no exclusion fencing around the stream or riparian area. A detailed description of the land cover and plant species composition of each site is provided below. The total precipitation in June, July, August and September of 2009 at Killarney (or a nearby weather station as described in Section 2.4) was 53.0 mm, 38.0 mm, 46.3 mm and 20.4 mm, respectively. The total precipitation in June, July, August and September of 2009 at Souris (or a nearby weather station as described in Section 2.4) was 52.4 mm, 74.8 mm, 44.9 mm and 72.2 mm, respectively. The province of Manitoba lies in the Prairie region of Canada and as such, neither site is deemed as rugged.

#### 2.1.1. Killarney

The pasture in Killarney possessed the following communities based on the dominant plant species: tame flats, tame slopes and tame uplands; native slopes complex; and forested upland and forested flat and riparian.

Tame flats, tame slopes and tame uplands community were comprised of grasslands dominated by introduced and exotic species including various mixtures of smooth brome (*Bromus inermis*), alfalfa (*Medicago sativa*), Kentucky bluegrass (*Poa pratensis*), clovers (*Trifolium* species), dandelion (*Taraxacum officinale*), intermediate wheatgrass (*Thinopyrum intermedium*) and/or orchardgrass (*Dactylis glomerata*). Thistles (*Cirsium* species) were also frequent on the flats.

The native slopes complex community in Killarney included steep south-facing slopes comprised of complex of native grasslands with patches of brush. The native grasslands contained mixtures of green needle grass (*Nasella viridula*), porcupine grass (*Hesperostipa spartea*), blue grama grass (*Bouteloua gracilis*), june grass (*Koeleria macrantha*), little bluestem (*Schizachyrium scoparium*), prairie dropseed (*Sporobolus heterolepis*), sedges (*Carex* species), and/or sages (*Artemisia* species). The brush patches present in this pasture contained mixtures of hawthorn (*Crataegus chrysocarpa*), chokecherry (*Prunus virginiana*), and/or Manitoba maple (*Acer negundo*), usually with an understory of sweet-scented bedstraw (*Galium triflorum*), sedges (*Carex* species) and/or smooth brome (*B. inermis*).

The main types of forest in Killarney were associated with the riparian flat and the more heavily treed uplands, which made up the forested upland, forested flat, and riparian communities. Maples (*A. negundo*) dominated the riparian woods, and oaks (*Quercus macrocarpa*) dominated the uplands, though in many areas the two mixed or graded into one another. Density of the woody vegetation was highly variable, from open shrub lands to dense closed canopies. Both types of forest had snowberry (*Symphoricarpos occidentalis*), chokecherry (*P. virginiana*), willow (*Salix* species), and hawthorn (*C. chrysocarpa*) for shrub cover.

#### 2.1.2. Souris

The historical management and the topography at the Souris site were far more complex than at the Killarney site, leading to a more complex mosaic of vegetative communities. The pasture in Souris was comprised of the following communities: tame hay, native grassland, and mixed upland; upland complex; open shrubby lowland; and moist depression, and oxbow meadow and wood.

The south ends of each paddock were dominated by smooth brome (*B. inermis*), quackgrass (*Thinopyrum repens*), and Kentucky bluegrass (*P. pratensis*). Native grasslands in the furthest east paddock contained mixtures of Kentucky bluegrass (*P. pratensis*), smooth brome (*B. inermis*), little bluestem (*S. scoparium*), green needle grass (*Nassella viridula*), porcupine grass (*H. spartea*), and bearded wheatgrass (*Elymus trachycaulus* var. *subsecundus*). Occasional shrubs that were present on these grassland areas included western snowberry (*S. occidentalis*), meadowsweet (*Spiraea alba*), willows (*Salix* species), hawthorn (*C. chrysocarpa*), wolf willow (*Elaeagnus commutata*), and rose (*Rosa* species). The northern part of each paddock was dominated by open grassland with a mixture of exotic and native species, including smooth brome (*B. inermis*), Kentucky bluegrass (*P. pratensis*), quackgrass (*T. repens*), pasture sage (*Artemisia frigida*), sedges (*Carex* species), and silverweed (*Argentina anserina*).

The largest vegetation type was the lowland complex adjacent to the stream. The complex was a mix of open meadow, shrub land and some trees. Typical herbaceous species in the open meadows were smooth brome (*B. inermis*), Kentucky bluegrass (*P. pratensis*), quackgrass (*T. repens*), narrow reedgrass (*Calamagrostis stricta*), sedges (*Carex species*), prairie cordgrass (*Spartina pectinata*), and silverweed (*A. anserina*). Frequent shrubs were willow (*Salix species*), dogwood (*Cornus sericea* subspecies *sericea*), rose (*Rosa* species), chokecherry (*P. virginiana*), and maple (*A. negundo*). Trees included mostly Manitoba maple (*A. negundo*) and willows (*Salix* species) with occasional aspen (*P. tremuloides*) and green ash (*F. pennsylvanica*). Older forest stands were frequent in the southern portions of the pasture.

A moist depression existed at the northwest portion of 3NOBARR, and was dominated by sedge (*Carex species*), Kentucky bluegrass (*P. pratensis*), and quackgrass (*T. repens*). Oxbows were common features on this landscape and transitioned from cattails in standing water to wetland grasses ringed by mature trees. Due to such variety, the oxbows were classed as one group of features. Common wetland herbs found in the depressional parts of these features were reed canary grass (*Phalaris arundinacea*), slough grass (*Beckmannia syzigachne*), sedges (*Carex* species), cattails (*Typha latifolia*), bulrushes (*Scirpus* species), narrow reedgrass (*C. stricta*), silverweed (*A. anserina*), quackgrass (*T. repens*), and Kentucky bluegrass (*P. pratensis*). Under trees and shrubs, upland species were more likely to dominate, such as quack grass (*T. repens*), Kentucky bluegrass (*P. pratensis*), smooth brome (*B. inermis*), and thistles (*Cirsium* species). Trees and shrubs that occurred around oxbows were willow (*Salix* species), meadowsweet (*S. alba*), western snowberry (*S. occidentalis*), rose (*Rosa* species), dogwood (*C. sericea* subspecies *sericea*), Manitoba maple (*A. negundo*), alder (*Alnus species*), raspberry (*R. idaeus*), and currant (*Ribes* species).

### 2.2. Pasture Management

The study was conducted over three, 28-day periods during the grazing season. At each site, three treatments were examined: no OSW nor barrier (1CONT), OSW with barrier (2BARR), and OSW without barrier (3NOBARR). The OSW system consisted of a submersible pump, a solar panel, battery, storage tank, and trough. Water was pumped from the stream into the storage tank, which filled the trough as the cattle drank. At each site, the OSW were situated north of the stream, with a tub containing supplementary minerals placed approximately 25 m from the OSW. At the Killarney site, the OSW was located approximately 60 m from the stream in 2BARR, while in 3NOBARR, the OSW was located approximately 120 m from the stream. At the Souris site, the OSW was located approximately 95 m from the stream in 2BARR, while in 3NOBARR, the OSW was located approximately 105 m from the stream. The OSW were placed to intercept the main flow of animal traffic.

**Figure 2:**
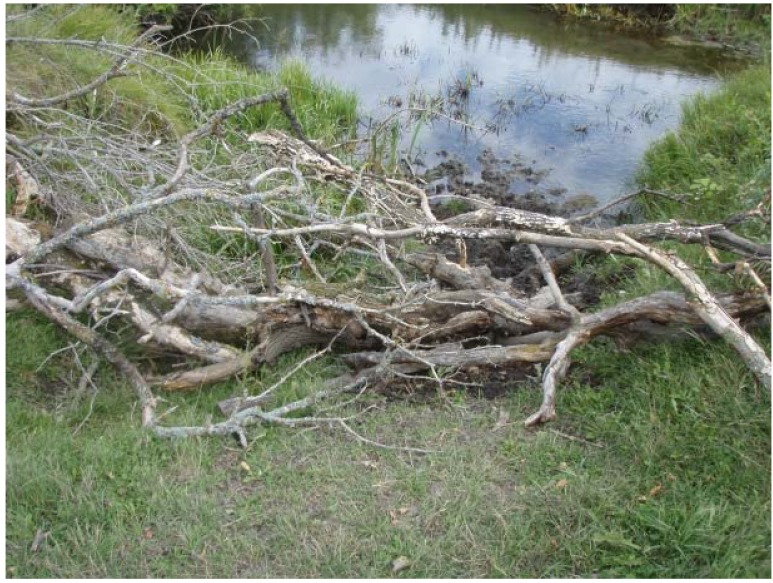
Example of a natural barrier constructed from deadfall (fallen trees and branches) placed at common watering and crossing locations in 2BARR.

In 2BARR, natural barriers, which consisted of deadfall (*i.e.*, fallen trees and branches; Figure 2) from the pasture, were placed across common watering and crossing areas on the north side of the stream. The intent was not to completely exclude cattle from the riparian area, but rather to encourage use of the watering system. Location of the barriers was determined before cattle were turned out at the beginning of the grazing season. Two established crossing points were left without barriers to allow access to the pasture on the south side of the stream. The riparian area was not fenced and the size of the stream was such that the cows were able to cross from one side to the other. The barriers were monitored throughout the season for evidence of hoof imprints or bare ground, and reinforced as required. New barriers were established if cattle appeared to be watering or crossing at new locations along the stream.

At the Killarney site, cow/calf pairs were turned out July 2, while at the Souris site, cow/calf pairs were turned out June 18, 2009. Cattle were allowed access to supplementary pastures at the Souris site to ensure that adequate biomass was available during the grazing season. The OSW were located so that cattle would pass them en route to the supplementary pastures.

Twenty-five cow/calf pairs were assigned to each treatment, with a total of 75 cow/calf pairs per site. In Killarney, the cows were Black Angus and the majority of the herd was first-calf heifers. Cows in Souris were of mixed breeds but consisted primarily of Charolais genetics. Both herds contained moderately-framed animals, which were well-adapted to the study area as they had previously grazed these sites. As a consequence of differences in reproductive performance between the two herds, calf numbers varied amongst treatments. At the Killarney site, the number of calves in 1CONT, 2BARR and 3NOBARR were 18, 26, and 22, respectively. There were 25 calves in each treatment at Souris. Midway through the grazing season in each year, one bull was assigned to each treatment.

To protect against horn flies, sucking lice, and biting lice, cows at both sites were treated with CyLence^®^ (Bayer Inc., Toronto, Canada). Salt blocks (Co-op Cobalt Iodized Salt, Federated Co-operatives Ltd., Saskatoon, Canada) and mineral (Co-op 3:1 Beef Cattle Mineral, Federated Co-operatives Ltd.) were available *ad libitum* in each pasture. Animal handling and care procedures in this study were carried out in accordance with the guidelines of the Canadian Council on Animal Care [15].

### 2.3. Cattle Location within the Pasture and Watering Location

A sample of cows was fitted with global positioning system (GPS) collars to monitor their location throughout the pasture in 2BARR and 3NOBARR (Table 1). Two models of collars were used: GPS3300LR Livestock GPS Collars (Lotek Wireless Inc., Newmarket, Canada) and GPS2200 Collars (Lotek Wireless Inc., Newmarket, Canada). The collars were programmed to record location fixes every five minutes and utilized for a minimum of 10 days in each period (Table 1).

**Table 1:** Collar distribution between treatments and number of days of data collection at each site and in each period.

Site	Period	Duration	Treatment	Number of Lotek collars utilized	Number of days of data obtained
**Killarney**	Period 1	02-Jul-09 to 29-Jul-09	2BARR	6	12
			3NOBARR	7	12
	Period 2	30-Jul-09 to 26-Aug-09	2BARR	5	10
			3NOBARR	6	10
	Period 3	27-Aug-09 to 23-Sep-09	2BARR	4	12
			3NOBARR	7	12
**Souris**	Period 1	18-Jun-09 to 15-Jul-09	2BARR	7	11
			3NOBARR	5	11
	Period 2	16-Jul-09 to 11-Aug-09	2BARR	6	12
			3NOBARR	7	12
	Period 3	12-Aug-09 to 9-Sep-09	2BARR	4	13
			3NOBARR	4	13

Positions from the collars were differentially corrected with N4 v.1. 2138 software (Lotek Engineering Inc., Newmarket, Canada) using base-station data downloaded from the Canadian Spatial Reference System Online database station in Winnipeg, Manitoba, located 192 km from the Killarney site and 227 km from the Souris site. With differential correction applied to the data, Moen *et al.* [16] reported the accuracy of Lotek GPS_1000 collars (Lotek Wireless Inc., Newmarket, Canada) to within 5 m, while Ganskopp and Johnson [17] reported the accuracy of Lotek GPS2200 Collars to within 2 m.

To identify when cattle were in the RP, buffers were created along the RP and around the OSW in ArcMap 10 (Environmental Systems Research Institute, Redlands, CA, USA). A 10 m buffer was created on either side of the center of the stream for the RP, while an 8 m buffer was created around the OSW. Data from the GPS collars was examined to identify each fix located within the boundary of the RP buffer and the OSW buffer. The term “RP” is used exclusively to describe the 10 m buffer created within the riparian area for the GPS collar data and for visual observation (VO) data, as described below.

Visual observations were conducted to record the watering location of cows fitted with GPS collars in 2BARR and 3NOBARR. Observations were recorded every five minutes and took place from dawn until dusk for four days of each period. Observational data was not collected at night, as previous research suggests that little activity occurs during the night [7,18]. Watering activity was recorded when cattle were in any of the following locations: stream (in stream or within one body length of the stream, approximately 2 m), RP (within five body lengths of the stream, approximately 10 m), or OSW (within four body lengths of the OSW, approximately 8 m). Sheffield *et al.* [10] used a similar method, where animal location was recorded as riparian when they were two body lengths from the center of the stream and as OSW when they were two body lengths from the edge of the OSW. The percentage of drinking events at the OSW or stream was calculated for each treatment and period.

### 2.4. Temperature-Humidity Index

Ambient temperature, relative humidity, and precipitation were recorded hourly using HOBO U30 Cellular data loggers (Onset Computer Corporation, Bourne, MA, USA) installed at both sites on July 15, 2009. When data from the HOBO U30 Cellular data loggers was not available (July 1 to July 15, 2009 of Period 1 in Killarney; all of Period 1 in Souris), temperature, relative humidity, and precipitation data were obtained from weather stations located near each site operated by the Manitoba Ag-Weather Program (MAFRI). The alternative stations were located 7.7 km and 13.2 km from the Killarney and Souris sites, respectively. The temperature-humidity index (THI), which is the basis for the Livestock Weather Safety Index (LWSI) [19], was used as a variable to analyze behavior in livestock, based on response to weather. The THI was calculated using the following calculation (1):

THI = (0.8 × T) + [(RH/100) × (T − 14.4)] + 46.4
(1)

where T is the temperature in °C and RH is the relative humidity expressed as a percentage [20].

Temperature and relative humidity data recorded on days when cattle were fitted with GPS collars were used to calculate THI, averaged for each three hour block of each day in each period.

### 2.5. Forage Biomass Measurements

To measure forage biomass in the upland and riparian area, a 0.25 m^2^ quadrat was randomly placed at nine locations in both riparian area and upland, with the upland samples collected in an M-pattern, as described in Ominski *et al.* [21]. The riparian samples were collected in a similar manner to the upland samples; however, instead of an M-shape, they were randomly thrown throughout the length of the riparian area. Standing grasses and forbs within the quadrat were clipped to a height of 3.75 cm stubble and then placed in labeled Delnet bags (DelStar Technologies, Inc., Austin, TX, USA). To determine dry mass content, sample bags were weighed, dried in a forced air oven at 60 °C for 48 h to a constant mass, and weighed again. Forage availability was sampled at the beginning of each period in the grazing season.

### 2.6. Animal Performance

Cows and calves were weighed on the first day of each period. Weigh days are identified as follows: day 1 of Period 1 (P1-D1), day 1 of Period 2 (P2-D1) and day 1 of Period 3 (P3-D1).

### 2.7. Statistical Analysis

Data from each cow fitted with a GPS collar, in each site, treatment, and period was grouped into eight, 3 h time blocks for analysis (0001 h to 0300 h, 0301 h to 0600 h, 0601 h to 0900 h, 0901 h to 1200 h, 1201 h to 1500 h, 1501 h to 1800 h, 1801 h to 2100 h, and 2101 h to 2400 h), as described in Porath *et al.* [22]. The number of GPS fixes (a fix was given every five minutes) within and outside the buffer area of the RP was determined for each 3 h period for each cow.

For each location (Killarney or Souris), the number of fixes measuring time spent in the RP in 2009 (*Y_ijklm_*) was examined using PROC GLIMMIX (SAS Institute Inc., Cary, NC, USA) with the following model (2):

*Y*_ijklm_ = µ + *t*_i_ + *b*_j_+ *p*_k_ + *tb*_ij_+ *tp*_ik_ + *bp*_jk_ + *tbp*_ijk_ + *d*_kl_ + *c*_ikm_+ *e*_ijklm_ (2)

where *t*_i_ is the effect of the i’th treatment, *b*_j_ is the effect of the j’th time block, *p*_k_ is the effect of the k’th period, with their two and three-way interactions denoted with letter combinations, all of these parameters were considered as fixed effects. Random effects included *d*_kl_ as the effect of the l’th day within the k’th period, and *c*_ikm_ is the effect of the m’th collar (*i.e.* cow) within the ik’th treatment and period, and the residual error, *e*_ijklm_. The interaction between treatment and time block was of particular interest since it provided an indication of whether or not cattle spent a different amount of time in the RP as the day progressed. Significance of factors was assessed using a type 1 error rate of 0.05. Using the GLIMMIX procedure, a binary distribution and a logit link function were assumed for the data. Results were presented as percentage of time in the RP relative to the total amount of time in a 3 h time block.

The THI data was analyzed with PROC MEANS (SAS Institute Inc.) to determine the mean, standard deviation (SD), minimum values, and maximum values for each period at each site and year.

Forage biomass in the riparian and upland pasture areas in each treatment and period were compared using t-tests (SAS Institute Inc.). Significance of differences was assessed using a type 1 error rate of 0.05.

Animal performance data, including cow and calf weights from each site, were analyzed separately. Repeated measures of analysis of variance were carried out in PROC MIXED (SAS Institute Inc.) for cows and calves with treatment and period included with the following model (3):

*Y*_ijk_ = µ + *t*_i_ + *d*_j_+ *td*_ij_+ *c*_ik_ + *e*_ijk_ (3)

where *t*_i_ is the effect of the i’th treatment, *d*_j_ is the effect of the j’th weigh date, *td*_ij_ is the treatment×weigh date effect of the i’th treatment and the j’th weigh date, all of these parameters were considered as fixed effects. Random effects included *c*_ik_ as the effect of the k’th animal within the i’th treatment and the residual error, *e*_ijk_. The interaction between treatment and period provided an indication of whether or not treatment affected animal performance differently as time passed during the grazing season. Significance of factors was assessed using a type 1 error rate of 0.05. Contrasts were developed to test hypotheses regarding treatment differences in different periods. If treatment differences were significant and positive, it was presumed that the availability of OSW was contributing to improved animal performance. However, if the treatment differences were significant and negative, it was presumed that the availability of OSW, or other factors such as forage biomass or climate reduced animal performance.

## 3. Results and Discussion

### 3.1. Cattle Location within the Pasture as Recorded by Global Positioning System Collars

Cattle in 2BARR spent less time in the RP than 3NOBARR cattle at the Killarney site in Period 1 (*p* = 0.0002) and Period 3 (*p* < 0.0001), as indicated in Table 2. This result is in agreement with the general expectation that cattle kept in an area with natural barrier (2BARR) would spend a lesser or similar amount of time in the RP compared with their counterparts kept in an area without any barrier (3NOBARR). Alternatively, cattle in 2BARR spent more time in the RP than 3NOBARR cattle at the Souris site in Period 1 (*p* < 0.0001), while the opposite occurred in Period 2, where 2BARR cattle spent less time in the RP than 3NOBARR cattle (*p* < 0.0001; Table 2).

**Table 2:** The mean percentage of time cattle fitted with GPS collars spent in the riparian polygon in Period 1, 2, and 3 throughout 2009.

	Killarney				Souris		
	Period 1	Period 2	Period 3		Period 1	Period 2	Period 3
2BARR	4.8 (0.8) ^1^	4.3 (0.8)	0.8 (0.2)		5.4 (1.2)	1.1 (0.3)	2.1 (0.5)
3NOBARR	7.6 (1.3)	5.3 (1.0)	4.0 (0.7)		1.3 (0.4)	2.8 (0.6)	1.7 (0.4)
*p*-Value	0.0002	0.1116	<0.0001		<0.0001	0.0002	0.5633

^1^ Percentage of time over 24 h with standard error in parentheses.

Without GPS data from 1CONT, it may not be possible to conclusively determine if the OSW was successful at decreasing the amount of time that cattle spent in the RP. However, the data does provide some indication of the efficacy of the natural barriers on deterring cattle from the RP. As cattle in 3NOBARR at the Killarney site spent a greater proportion of time in the RP throughout all three periods, it suggests that the implementation of the barrier in 2BARR was effective in deterring cattle from remaining in the RP. However, these results are inconsistent with Souris, where cattle in 2BARR were found to spend a greater proportion of time in the RP in Periods 1, while cattle in 3NOBARR spent a greater proportion of time in Period 2.

At the Souris site, the grazing areas for 3NOBARR, 2BARR and 1CONT were 39.2, 26.3 and 30.4 ha, respectively. The size of 3NOBARR was 49% bigger than 2BARR. One criterion for deciding the size of grazing area at the beginning of the study was carrying capacity of the pasture; for example, the forage biomass of the upland pasture in 2BARR was 51% higher than 3NOBARR. This variation in the allocated size of pasture may have influenced the behavior of the animals and thereby caused the observed discrepancy at Souris. In the later stage of the grazing season, the animals accessed supplementary pastures (of roughly similar sizes) and that might have changed biomass availability. Cattle at the Souris site spent less time in RP than did the cattle at the Killarney site. The cows at the Souris site were generally older than their counterparts at Killarney. In a study conducted in Oregon, DelCurto *et al.* [23] reported that older cows spent less time in riparian areas than two-year old cows which was attributed to greater familiarity of the terrain.

These mixed results may also indicate that natural and constructed barriers such as those utilized in the current study, may not be sufficient to consistently discourage cattle from spending time within the RP. More specifically, the barriers may not have been large enough to deter cattle from the RP, and if the barrier did serve as a deterrent, cattle may have moved further along the RP to an area without a barrier. It has been suggested that dense or thorny hedges which are unpalatable to cattle (hawthorns or rose bushes) may serve as effective barriers [14]. In the present study, using natural deadfall was not consistently successful as the barriers were also time consuming to maintain and cattle seemed to maneuver through the barrier if they entered the stream from the opposite side. Examining the effectiveness of different types of barrier material, such as hedges or strategic fences, may serve to improve the effectiveness of barriers in deterring cattle from watering at the stream, thus encouraging usage of the OSW. The economic implication of the time required to maintain the structures should also be taken into account as it could offset the benefit of using inexpensive materials to construct the barriers. The use of natural barriers, particularly fallen trees, may only be feasible in areas of forested to partly forested riparian areas. In areas with only scattered riparian forests, as is common in arid areas, the use of natural barriers may not be feasible. Other strategies such as placing salt/mineral blocks next to OSW (as was done in this study) is also expected to further attract cattle into the uplands.

**Figure 3:**
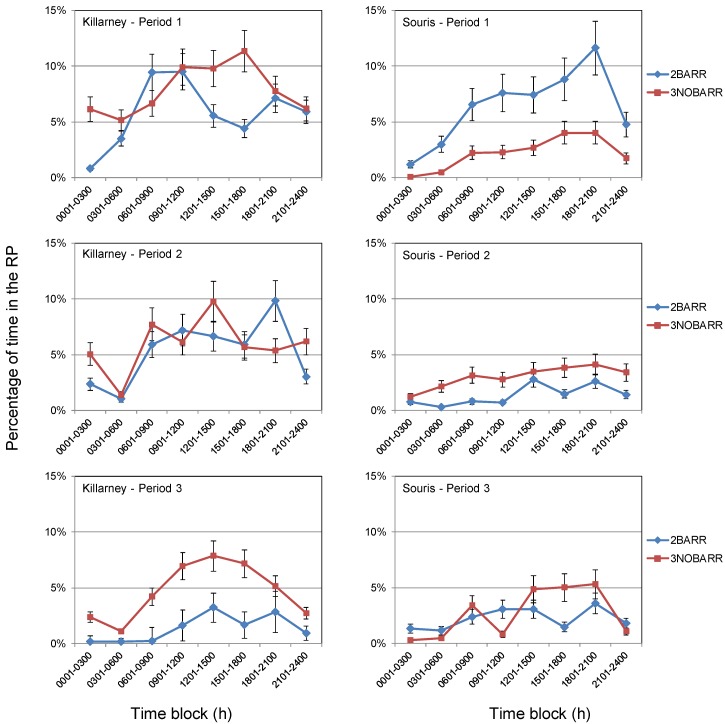
Percent of time cattle at the Killarney and Souris sites spent in the riparian polygon for each time block during Period 1, 2, and 3.

A consistent pattern regarding time spent in the RP was evident as cattle spent the lowest percentage of time in the RP overnight, but a greater percentage of time in the RP as the day progressed, as depicted in Figure 3. Cattle in 2BARR at the Killarney site spent the greatest percentage of time within the RP from 0901 h to 1200 h, 1801 h to 2100 h, and 1201 h to 1500 h in Periods 1, 2, and 3, respectively. Cattle in 3NOBARR spent the greatest percentage of time in the RP from 1501 h to 1800 h, 1201 h to 1500 h, and 1201 h to 1500 h in Periods 1, 2, and 3, respectively. Cattle in 2BARR appeared to begin the day (0001 to 0600) further from the stream than cattle in 3NOBARR, but the disparity diminished in the first two periods as the day progressed.

Cattle in 2BARR at the Souris site spent the greatest percentage of time within the RP from 1801 h to 2100 h, 1201 h to 1500 h, and 1801 h to 2100 h in Periods 1, 2, and 3, respectively (Figure 3). Cattle in 3NOBARR spent the greatest percentage of time the RP from 1501 h to 2100 h, 1801 h to 2100 h, and 1801 h to 2100 h in Periods 1, 2, and 3, respectively.

The percentage of time in the RP in each time block fluctuated between periods. However, irrespective of the presence of barriers, a general trend was apparent in that the percentage of time that cattle spent in the RP was limited during the night and early morning (0001 h to 0600 h), increased throughout the late morning (0901 h to 1200 h), remained high throughout the afternoon, and decreased again during the evening (2101 h to 2400 h). This trend is similar to that reported by Gillen *et al.* [24] and Porath *et al.* [22] and follows a similar pattern to that observed for daily temperature. As the temperature increased during the day, cattle spent an increased percentage of time in the RP. Cattle actively seek shade during the hottest part of the day [25]; therefore, cattle may have spent a greater percentage of time in the RP during the afternoon as they were seeking shade for relief from heat.

In addition to the time of the day effects on the percentage of time cattle spent in the RP, seasonal impacts on distribution were also apparent. The percentage of time 2BARR and 3NOBARR cattle spent within the RP at the Killarney site declined as the grazing season progressed (Table 2). Cattle in 2BARR spent a greater percentage of time within the RP early in the grazing season at the Souris site, and spent less time as the season progressed.

Cattle at the Killarney site spent more time in the RP in the first two periods, potentially grazing riparian vegetation heavily in the earlier part of the season and moving into the upland pasture in search of more vegetation as the season progressed. The authors cannot conclusively explain the unusual pattern in Souris, but speculate that the cattle were spending more time in the supplemental pastures, allowing for recovery of the main pastures. Porath *et al.* [22] hypothesized that due to the higher rate of forage utilization in the riparian area during the early part of the grazing season, cattle had to travel further from the stream later on in the season in order to find adequate vegetation, which is similar to the behavior observed in 2BARR and 3NOBARR at the Killarney site, and in 2BARR at the Souris site.

### 3.2. Watering Location as Recorded by Visual Observations

**Figure 4:**
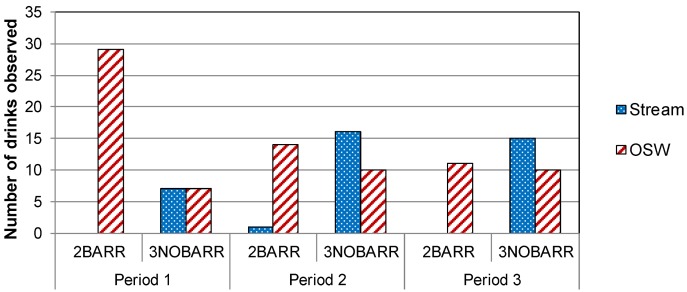
Watering location used by collared cows over four days at the Killarney site during Period 1, 2, and 3.

At the Killarney site, 100%, 93%, and 100% of observed watering events for the collared cows in 2BARR occurred at the OSW in Periods 1, 2, and 3, respectively (Figure 4). In 3NOBARR, 50%, 38%, and 40% of observed watering events for collared cows occurred at the OSW in Periods 1, 2, and 3, respectively.

**Figure 5:**
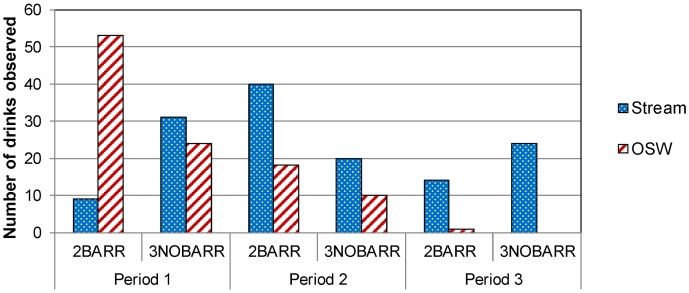
Watering location used by collared cows over four days at the Souris site during Period 1, 2, and 3.

At the Souris site, 85%, 31%, and 7% of observed watering events for the collared cows in 2BARR occurred at the OSW in Periods 1, 2, and 3, respectively (Figure 5). In 3NOBARR, 44%, 33%, and 0% of observed watering events for collared cows occurred at the OSW in Periods 1, 2, and 3, respectively. The very low percentage of observed watering events at OSW in Period 3 may be partly attributable to the fact that the animals at the Souris site had access to supplementary pastures later in the season, which was comparatively far from the OSW.

Many researchers have found that cattle prefer to water at an OSW compared to a stream when an OSW is available [8,10,13]. In contrast, our results are similar to Bagshaw *et al.* [26], who found that cattle watered at the OSW, but the availability of the OSW did not decrease watering at the stream or time spent in the riparian area. Our results indicate that cattle did water at the OSW; however, they did not use it exclusively. Watering at the stream continued despite the availability of OSW, with the exception of 2BARR at the Killarney, where cattle were consistently observed watering at the OSW.

The observed differences in watering behavior may be attributed to several factors. Veira and Liggins [13] suggested that dry, firm, and level soil surrounding the OSW provided better footing than watering locations along the stream. In addition, the location of the OSW may also be a critical factor which affects usage. Sheffield *et al.* [10] compared two periods, a pre-BMP period with access to stream only and a post-BMP period with access to stream and OSW (with the OSW located adjacent to the stream), to examine OSW usage in three pastures which were 14.2 ha, 16.6 ha, and 22.3 ha in size. Their results indicated that when given the choice, cattle watered at the OSW 92% of the time compared to the stream. Bryant [27] examined the impact of placement of an OSW in a 344.8 ha pasture, where the OSW was located 1.5 km upslope from the stream. They observed that exclusive use of the OSW or the stream depended on proximity of cattle to either source within the pasture. This implies that the distance that cattle must travel to their water source will significantly influence the likelihood of usage of an alternative water source, such as an OSW. Pasture size in the current study ranged from 21.0 ha to 39.2 ha with an OSW located exclusively on the north side of the stream in 2BARR and 3NOBARR. Although cattle did water at the OSW, they continued to water at the stream as well, using the OSW as a supplemental source rather than as the primary water source. As observed by Bryant [27], cattle in the current study likely selected their watering location based on proximity. If cattle were a substantial distance from the OSW, such as the south side of the stream, they were likely unmotivated to travel the distance to the OSW when they could access water from the closer stream source. The size of the pastures in the current study may not be large enough to effectively analyze the impact of placement of an OSW and provide conclusive results. Installing an OSW on both sides of the stream would ensure that cattle had easy access to the OSW, without having to travel further or cross the stream, potentially increasing usage of the OSW. However, installing multiple OSW within a pasture is costly, thus its feasibility is limited.

In some periods, the total number of watering events recorded for individual cows is less than the average number of one to four drinks per day, as reported by Hafez and Bouissou [28]. Some watering events may have been missed by the observers given the topography and the prominent bush present at the site. Pandey *et al.* [29] found that observations may be missed due to observer fatigue, or from observer proximity effects on livestock.

Placement of OSW may significantly impact the grazing behavior of animals, and thereby the vegetation cover of riparian areas. Rigge *et al.* [30] investigated the optimal placement of OSW using satellite imagery time series of western South Dakota mixed-grass prairie. Their findings suggest that placement of OSW between 200 m and 1250 m from streams provided optimal results in the sites studied. In the present study, the OSW were placed 60 m–120 m from the streams. As optimal placement of OSW was not one of the factors under investigation, site-specific investigation of optimal placement of OSW in commercial scale pastures that contain riparian areas is warranted.

### 3.3. Temperature-Humidity Index (THI)

Heat stress, as measured by THI, may be classified as follows: THI ≤ 74, normal; 74 < THI < 79, alert; and 79 ≤ THI < 84, danger; and THI ≥ 84, emergency [19]. Other studies using cow/calf pairs have lowered the threshold for heat stress to account for the heat produced by the lactating cow, thus identifying cattle as heat stressed when the THI exceeds 72 [31,32]. The mean, standard deviation (SD), minimum, and maximum THI values were calculated for each 3 h time block in a 24 h period when cattle were fitted with GPS collars (Table 3).

Mean THI typically increased in the morning, remained highest between 1200 h and 1800 h, and then decreased in the evening. In the current study, the mean THI does not exceed 72 at any point in the day during Period 1, 2, and 3 in 2009. However, the maximum THI exceeded 72 during the 3 h block on a number of days at each site. At the Killarney site, the THI did not exceed 72 in Periods 1 and 2, but did for 7 out of 12 days in Period 3. At the Souris site, the THI of 72 was exceeded five out of 11 days, 5 out of 12 days, and 2 out of 13 days in Period 1, 2, and 3, respectively.

Cattle may be attracted to riparian areas to seek relief when heat stressed [18,25]. Franklin *et al.* [32] found that cattle in the Georgia Piedmont region spent a greater proportion of time in the riparian area and the stream when the THI was high (72–84). The overall mean THI in the study by Franklin *et al.* [32] was significantly higher (75) than that observed at Killarney and Souris, which ranged from 60 to 64.

**Table 3:** Temperature-humidity index (THI) by time block and period over days when cattle were fitted with GPS collars in Killarney and Souris.

Site	Period 1					Period 2						Period 3			
Time Block	Mean	SD	Min	Max		Mean		SD	Min	Max		Mean	SD	Min	Max
***Killarney***															
0001–0300	53	4	47	60			54	6	46	64		58	5	48	63
0301–0600	52	4	44	58		54		7	44	62		57	6	46	65
0601–0900	57	3	52	62		56		4	49	63		58	4	52	62
0901–1200	63	3	59	67		62		2	59	67		68	4	61	74
1201–1500	66	3	62	69		65		3	61	71		71	5	64	77
1501–1800	66	3	59	69		66		3	58	71		71	5	63	77
1801–2100	64	3	57	68		63		3	56	68		66	5	58	72
2101–2400	57	4	48	64		57		4	50	65		60	6	51	67
Overall mean	60	3	44	69		60		4	44	71		64	5	46	77
***Souris***															
0001–0300	59	4	51	65		56		7	45	66		56	7	42	66
0301–0600	57	5	51	65		53		6	43	62		54	8	39	63
0601–0900	61	4	55	67		58		4	52	64		56	5	46	64
0901–1200	67	4	61	73		68		2	65	71		63	5	54	72
1201–1500	69	3	64	74		71		3	67	74		67	5	57	74
1501–1800	70	3	65	75		71		3	68	75		68	5	57	76
1801–2100	68	3	64	73		69		3	64	74		65	5	57	74
2101–2400	62	3	56	66		61		5	51	68		58	6	46	68
Overall mean	64	4	51	75		63		4	43	75		61	6	39	76

It is possible that the THI observed at Killarney and Souris was not consistently high enough to have a significant impact on cattle behavior [26]. Furthermore, as previously mentioned, the THI increased between 1200 h and 1800 h, and then decreased the remainder of the day, indicating that cattle at both Killarney and Souris experience night cooling. Previous research has demonstrated that cattle are dependent on cooler night temperatures as it allows them to dissipate the heat they have accumulated throughout the day [33,34]. Without night cooling as a means to dissipate heat accumulated throughout the day, cattle may be more attracted to the riparian area to seek out shade or enter the stream to find relief from heat. Cattle in the current study experienced night cooling at both locations, and as such, were presumably able to dissipate the heat they accumulated throughout the day, and thus may not have relied on the riparian area for relief from heat.

### 3.4. Forage Biomass

Forage biomass at the Killarney site was significantly greater in the riparian area than the upland in 3NOBARR (*p* < 0.0001) during Period 1, as well as 1CONT (*p* = 0.01) and 2BARR (*p* = 0.0002) in Period 2 (Table 4). The amount of forage biomass available in the upland decreased as the grazing season progressed in 1CONT, 2BARR, and 3NOBARR. However, the amount of forage biomass in the riparian area remained consistent throughout the grazing season in 1CONT, while forage biomass decreased in 2BARR and 3NOBARR. The reduction of the amount of forage biomass in the riparian area of 3NOBARR in Period 3 was relatively large. Although it may be speculated that this is the effect of the natural barrier (partial exclusion), a comparative decline in forage biomass was not observed in 1CONT. From the biomass values (2030.8 kg/ha in 1CONT and 5124.8 kg/ha in 3NOBARR), the riparian area in 3NOBARR might have appeared more attractive for the animals than riparian area in 1CONT. At the Souris site, the accumulation of riparian forage biomass in the second period was sizeable for the 3NOBARR (compared to other systems). This might again be attributable to the size of the grazing area.

**Table 4:** Forage biomass (kg/ha) in the riparian and upland areas at the Killarney and Souris sites.

Site	Period	Treatment	Forage Biomass (kg/ha)		
			Upland	Riparian	Significance of parameters
**Killarney**	**1**	1CONT	1565.5	2030.8	0.29
		2BARR	1806.0	2623.6	0.09
		3NOBARR	2070.5	5124.8	<0.0001
	**2**	1CONT	1045.9	2198.2	0.01
		2BARR	468.9	1750.8	0.0002
		3NOBARR	1408.8	1289.7	0.70
	**3**	1CONT	985.2	2069.6	0.21
		2BARR	849.5	935.7	0.80
		3NOBARR	1326.8	1317.4	0.99
**Souris**	**1**	1CONT	799.8	622.8	0.48
		2BARR	780.5	1163.4	0.22
		3NOBARR	517.7	792.8	0.13
	**2**	1CONT	453.5	325.4	0.42
		2BARR	589.0	1277.3	0.04
		3NOBARR	356.2	1381.6	0.01
	**3**	1CONT	760.0	997.0	0.42
		2BARR	845.2	1442.5	0.22
		3NOBARR	472.1	1676.4	0.003

Forage biomass at the Souris site was significantly greater in the riparian area than the upland in 2BARR (*p* = 0.04) and 3NOBARR (*p* = 0.01) during Period 2, as well as 3NOBARR (*p* = 0.003) in Period 3 (Table 4). The amount of forage biomass available in the upland pasture decreased as the grazing season progressed in 1CONT and 3NOBARR, while forage biomass increased in 2BARR. However, the amount of forage biomass in the riparian area increased over the grazing season in 1CONT, 2BARR, and 3NOBARR. Forage biomass was greater in the riparian area compared to upland pasture for the majority of observations from both sites.

The types of vegetation found in Manitoba are typically cool-season or warm-season species. Cool-season species grow early in the spring when more moisture is available, before becoming dormant during the heat of summer. Warm-season species break dormancy later in spring and are able to continue to grow throughout the hot, dry summer months [35]. Although percent cover of individual grass species was not recorded, the majority of the dominant grass species are cool season, indicating that forage biomass would likely decrease throughout the upland pasture as summer temperatures increased. Forage within riparian areas tends to remain more productive throughout the grazing season as result of the higher water table adjacent to the river or stream [6]. Results from a study conducted in the neighboring province of Ontario indicated that the nutritional quality and botanical composition of diverse grass-legume pasture varied during the grazing season [36]. According to Marshall *et al.* [36], the crude protein content began to increase during July through to the end of the grazing season and surpassed levels seen in the beginning of the grazing season (May) while fiber content increased from May to late June and were lowest in the cooler spring and fall months. These fluctuations are partly attributable to the varying growth patterns of complex grass-legume mixtures and climatic factors.

### 3.5. Animal Performance

As a consequence of differences in animal population, site topography, and precipitation, the weight gain of cows and calves in the two treatments with OSW (2BARR and 3NOBARR) was compared to that for cows and calves in the control treatment (1CONT) at each site. For each class of animal (calves and cows), the significance of treatment, period, and the associated interaction at both sites are provided in Table 5.

**Table 5:** The effect of treatment, period, and their interaction on weight of calves and cows at the Killarney and Souris sites (P-values for each effect from the ANOVA).

Site	Calves/cows	Significance of parameters		
		Treatment	Period	Treatment × Period
**Killarney**	Calves	0.5523	<0.0001	0.1088
	Cows	0.9032	<0.0001	<0.0001
**Souris**	Calves	0.1454	<0.0001	0.0177
	Cows	0.8891	<0.0001	<0.0001

**Figure 6:**
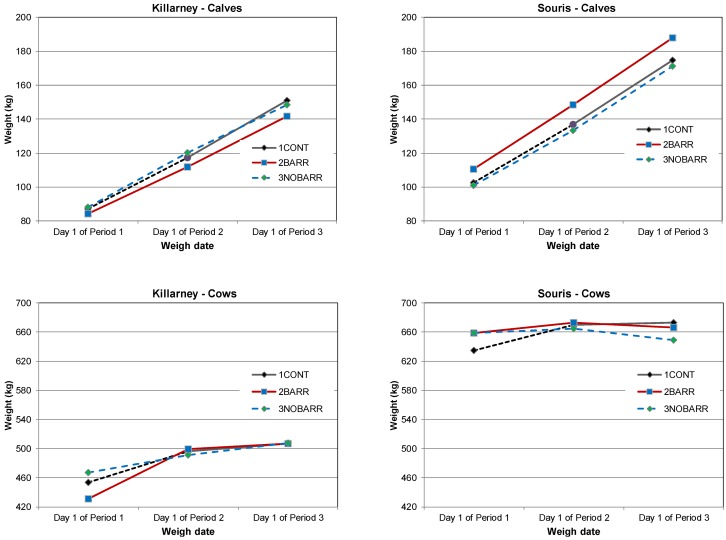
Average weights of calves and cows at the Killarney and Souris sites.

The initial weight of the calves and cows recorded on P1-D1 was used as a reference for treatment differences in subsequent periods, as treatment effects, if important, should appear over time. Treatment differences at the end of the first and second period were compared to differences present at the beginning of the first period (which may be present as a result of random chance). There was no change in weight gain in 2BARR calves from P1-D1 to P2-D1 (*p* = 0.4042) compared to weight change realized by 1CONT calves at the Killarney site (Table 6). However, significant weight change (*p* = 0.0242) amongst 2BARR calves did occur from P3-D1 relative to P1-D1, with lower weight gain in 2BARR compared to 1CONT calves (Figure 6).

Cows in 2BARR at Killarney had significantly greater weight gain compared to 1CONT cows in P2-D1 relative to P1-D1 (*p* < 0.0001) and P3-D1 relative to P1-D1 (*p* = 0.0001). The positive differences in weight gain between 2BARR cows and 1CONT cows, and the negative differences between 3NOBARR cows and 1CONT suggest the presence of the OSW had an impact on weight gain (Table 6). However, as the variation in weights was no longer apparent as the season progressed (Figure 6), the observed differences in weight gain cannot be only attributed to the OSW.

**Table 6:** Change in weight of calves and cows over the grazing season at the Killarney and Souris sites.

Site	Calves/cows	Treatment compared	Weigh dates	Significance of parameters	Weight change (kg) ^1^	Weight change in favour of OSW
Killarney	Calves	2BARR *vs.* 1CONT	P2-D1 *vs.* P1-D1	0.4042	−2.6 (3.1)	−
		2BARR *vs.* 1CONT	P3-D1 *vs.* P1-D1	0.0242	−6.2 (2.7)	No
		3NOBARR *vs.* 1CONT	P2-D1 *vs.* P1-D1	0.4821	2.0 (2.8)	−
		3NOBARR *vs.* 1CONT	P3-D1 *vs.* P1-D1	0.2469	−3.2 (2.8)	−
	Cows	2BARR *vs.* 1CONT	P2-D1 *vs.* P1-D1	<0.0001	24.6 (5.7)	Yes
		2BARR *vs.* 1CONT	P3-D1 *vs.* P1-D1	0.0001	20.3 (5.1)	Yes
		3NOBARR *vs.* 1CONT	P2-D1 *vs.* P1-D1	0.0003	−18.9 (5.1)	No
		3NOBARR *vs.* 1CONT	P3-D1 *vs.* P1-D1	0.0058	−14.2 (5.1)	No
Souris	Calves	2BARR *vs.* 1CONT	P2-D1 *vs.* P1-D1	0.0937	3.7 (2.2)	−
		2BARR *vs.* 1CONT	P3-D1 *vs.* P1-D1	0.0183	5.3 (2.2)	Yes
		3NOBARR *vs.* 1CONT	P2-D1 *vs.* P1-D1	0.4326	−1.7 (2.2)	−
		3NOBARR *vs.* 1CONT	P3-D1 *vs.* P1-D1	0.3966	−1.9 (2.2)	−
	Cows	2BARR *vs.* 1CONT	P2-D1 *vs.* P1-D1	0.0010	−21.3 (6.3)	No
		2BARR *vs.* 1CONT	P3-D1 *vs.* P1-D1	<0.0001	−31.2 (6.4)	No
		3NOBARR *vs.* 1CONT	P2-D1 *vs.* P1-D1	<0.0001	−29.4 (6.4)	No
		3NOBARR *vs.* 1CONT	P3-D1 *vs.* P1-D1	<0.0001	−48.0 (6.4)	No

^1^ Average weight change between weigh dates with standard error in parentheses.

There was no change in weight gain in 2BARR calves from P1-D1 to P2-D1 (*p* = 0.0937) compared to weight change realized by 1CONT calves at the Souris site. However, significant, positive weight change (*p* = 0.0183) in 2BARR calves did occur from P3-D1 relative to P1-D1 (Table 6). Cows in 2BARR had significantly lower weight gain compared to 1CONT cows in P2-D1 relative to P1-D1 (*p* = 0.0010) and P3-D1 relative to P1-D1 (*p* < 0.0001), as depicted in Figure 6. Similarly, 3NOBARR cows had significantly lower weight gain compared to 1CONT cows in P2-D1 relative to P1-D1 (*p* < 0.0001) and P3-D1 relative to P1-D1 (*p* < 0.0001). The negative differences in weight gain between 2BARR and 3NOBARR cows relative to 1CONT cows may suggest the availability of OSW, or other factors such as forage biomass or climate reduced animal performance. However, in view of differences in the size of the grazing areas, forage biomass and the use of supplementary pastures at the Souris site, it was very unlikely that the presence of OSW negatively affected animal performance. Body condition scores (BCS) of all treatment groups fell in between 3 and 3.5 (as per the 5-point BCS system) indicating the cows maintained BCS that would reasonably support reproductive function under western Canadian conditions.

Research by Porath *et al.* [22] showed that access to OSW and salt improved the average daily gain (ADG) of cows and calves as compared to those animals that did not have access to OSW or salt. Our results indicated that OSW may improve gains but that improvement is not consistent throughout the grazing season. In some instances, the presence of an OSW may act in favor of animal performance; however, other factors, such as management, available forage biomass, and temperature also impact gain.

## 4. Conclusions

This study provides novel information regarding the effectiveness of OSW with or without barriers in large-scale pastures located in temperate climates. Results indicate that cattle watered at the OSW when available, but they did not use the OSW exclusively. When comparing the percentage of time that cattle spent in the riparian area with or without barriers, the presence of the natural barriers did not consistently prevent cattle from watering at the stream though the data provided some indication of the efficacy of the barriers on deterring cattle from the riparian area.

Overall, this study demonstrates the continued need for multidisciplinary approaches to determine the effectiveness of the use of OSW as a recommended BMP. Both the impact on livestock behavior and performance, as well as to environmental sustainability, must be considered and evaluated as the effectiveness of OSW depends on a number of factors such as the site location, site topography, climate, and the prior experience of cattle within the site. Further research is necessary to determine complementary management strategies, such as the implementation of shade structures adjacent to the OSW that will increase cattle usage. In addition, future studies should also evaluate the impact of OSW on riparian health. It is suggested to conduct these studies over a longer period of time as riparian areas may take multiple years to regenerate, as well as to capture the effect of year-to-year variation in environmental conditions. Further research is also warranted to assess the effects of distance of OSW from RP and specific site characteristics of the OSW site on cattle behavior and performance.
